# Large-scale DCMs for resting-state fMRI

**DOI:** 10.1162/NETN_a_00015

**Published:** 2017-01-01

**Authors:** Adeel Razi, Mohamed L. Seghier, Yuan Zhou, Peter McColgan, Peter Zeidman, Hae-Jeong Park, Olaf Sporns, Geraint Rees, Karl J. Friston

**Affiliations:** The Wellcome Trust Centre for Neuroimaging, University College London, London, United Kingdom; Monash Biomedical Imaging and Monash Institute of Cognitive and Clinical Neurosciences, Monash University, Clayton, Australia; Department of Electronic Engineering, NED University of Engineering and Technology, Karachi, Pakistan; Cognitive Neuroimaging Unit, Abu Dhabi, United Arab Emirates; CAS Key Laboratory of Behavioral Science and Magnetic Resonance Imaging Research Center, Institute of Psychology, Chinese Academy of Sciences, Beijing, China; Huntington’s Disease Centre, Institute of Neurology, University College London, London, United Kingdom; Department of Nuclear Medicine and BK21 PLUS Project for Medical Science, Yonsei University College of Medicine, Seoul, Republic of Korea; of Psychological and Brain Sciences, Indiana University, Bloomington, Indiana; Institute of Cognitive Neuroscience, University College London, London, United Kingdom.

**Keywords:** Dynamic causal modeling, Effective connectivity, Functional connectivity, Resting state, fMRI, Graph theory, Bayesian inference, Large-scale networks

## Abstract

This paper considers the identification of large *directed* graphs for resting-state brain networks based on biophysical models of distributed neuronal activity, that is, *effective connectivity*. This identification can be contrasted with *functional connectivity* methods based on symmetric correlations that are ubiquitous in resting-state functional MRI (fMRI). We use spectral dynamic causal modeling (DCM) to invert large graphs comprising dozens of *nodes* or regions. The ensuing graphs are directed and weighted, hence providing a neurobiologically plausible characterization of connectivity in terms of excitatory and inhibitory coupling. Furthermore, we show that the use of Bayesian model reduction to discover the most likely sparse graph (or model) from a parent (e.g., fully connected) graph eschews the arbitrary thresholding often applied to large symmetric (functional connectivity) graphs. Using empirical fMRI data, we show that spectral DCM furnishes connectivity estimates on large graphs that correlate strongly with the estimates provided by stochastic DCM. Furthermore, we increase the efficiency of model inversion using functional connectivity *modes* to place prior constraints on effective connectivity. In other words, we use a small number of modes to finesse the potentially redundant parameterization of large DCMs. We show that spectral DCM—with functional connectivity priors—is ideally suited for directed graph theoretic analyses of resting-state fMRI. We envision that directed graphs will prove useful in understanding the psychopathology and pathophysiology of neurodegenerative and neurodevelopmental disorders. We will demonstrate the utility of large directed graphs in clinical populations in subsequent reports, using the procedures described in this paper.

## INTRODUCTION

Dynamic causal modeling is a Bayesian framework that allows one to make inferences about the causal (directed) interactions between the nodes (e.g., brain regions) of a coupled system; namely, effective connectivity (Razi & Friston, 2016). Effective connectivity contrasts with correlation-based functional connectivity that is inherently undirected. Usually, DCM is used to test hypotheses about subgraphs or brain networks that contain a relatively small number of nodes that are of specific interest in the context of an experimental manipulation. However, in recent years, there has been a marked increase in research that combines resting-state fMRI with large-scale, big-data network analyses (Nakagawa, Jirsa, Spiegler, McIntosh, & Deco, 2013; Smith et al., 2013; Sporns, 2014). Resting-state fMRI allows one to study connectivity in the brain through the acquisition of fMRI data, as participants lie at rest in an MRI scanner. Analyzing large-scale resting-state functional brain networks using graph theory has become a popular approach to these data (for reviews see Bullmore & Sporns, 2009; Fornito, Zalesky, & Breakspear, 2015). Graph theoretic characterizations generally rest upon the statistics of the edges, using descriptive statistics like degree, path lengths and clustering coefficients, or community detection methods to extract densely coupled clusters or modules (Rubinov & Sporns, 2010; Sporns & Betzel, 2016). There are several international efforts in place to characterize brain connectivity and its variability in healthy adults. For example, the Human Connectome Project has collected a rich set of large data (> 1,200 participants) and the UK Biobank project aims to scan 100,000 participants, using several neuroimaging modalities—including resting-state fMRI—to compute functional and effective connectivity and diffusion weighted MRI for estimating anatomical connectivity. While the structural connectome can be characterized using a variety of computational approaches including probabilistic fiber tracking, resting-state fMRI has a complementary role in characterizing the “functional connectome”—through a detailed mapping of functional integration. Unlike structural networks, functional networks refer to statistical constructs that express substantial variability on short timescales—in response to either external inputs or endogenous (spontaneous) activity—as in the case of resting-state fMRI. This means that functional connections are time and context sensitive, unlike anatomical connectivity which is conserved over shorter timescales. Crucially, these functional links exist without mandating any direct (monosynaptic) connection between neuronal populations; for example, polysynaptic connections that mediate functional coupling vicariously, such as through transitive closure.

*Functional connectivity* reflects the statistical dependencies between spatially remote neurophysiological events (Razi & Friston, 2016). These correlations are inherently undirected and—as the statistical dependencies are “model-free”—do not support any inference about (directed) causal interactions between neuronal systems. In contrast, *effective connectivity* measures the directed (causal) influence of one neural system over another using a model of neuronal interactions that best explains the observed signal fluctuations (Breakspear, 2004; Friston, Frith, Liddle, & Frackowiak, 1993). Dynamic causal modeling (Friston, Harrison, & Penny, 2003) is the most widely adopted framework to estimate effective connectivity. The key concept underlying DCM is to treat the brain as a nonlinear dynamic system that accepts multiple inputs and produces multiple outputs (i.e., MIMO model). This neuronal MIMO model is augmented with a regionally specific hemodynamic forward model that describes the mapping from neuronal activity to observed (fMRI) responses. Together, the neuronal and observation model comprise a full generative model.

This paper demonstrates that a recent variant of DCM (Friston, Kahan, Biswal, & Razi, 2014)—namely, spectral DCM that was designed to model resting-state fMRI—can be used to invert large-scale graphs. We show that spectral DCM can handle graphs comprising dozens of nodes and may therefore contribute to a mechanistic understanding of large-scale connectivity. Traditionally, DCM has been used to test competing hypothesis that embody a priori hypotheses about networks comprising only a few regions. Several competing hypotheses (that constitute a model space) are specified in the form of subgraphs, which are then compared using Bayesian model selection. However, increasing the number of regions or nodes in a DCM presents some challenges. For example, the number of extrinsic (between-node) connections or edges increases with the square of the number of nodes. This can lead to models with an enormous number of free parameters and profound conditional dependencies among the parameters. Furthermore, the computational time required to invert these models grows exponentially with the number of free parameters. Because stochastic DCM has to estimate both hidden (neuronal) states and parameters, it is computationally more intensive and—in its current form—unable to invert models with more than 10 nodes within pragmatic time frames. In contrast, spectral DCM has a much lower computational complexity and is ideally suited to invert large-scale models comprising in the order of 32 to 64 brain regions.

In this work, we used empirical data to invert graphs comprising 36 brain regions and establish the construct validity of the ensuing parameter estimates using two inversion schemes: stochastic DCM that inverts models in the time domain and spectral DCM, which is based on inversion in the frequency domain. Razi, Kahan, Rees, and Friston (2015) established *in silico* construct validation of spectral DCM against stochastic DCM. These analyses showed that spectral DCM was not only computationally efficient but also more accurate in terms of root mean squared error and sensitivity to group differences. In this paper, we use empirical data to show that spectral DCM is a computationally viable method for inferring large directed graphs of effective connectivity.

This paper comprises three sections. The first describes the requisite background for dynamical causal modeling of resting-state fMRI data and the model reduction procedures that can be used to place prior constraints on large networks for efficient inference. We then present the empirical data, attending model specification, and inversion procedures. In the subsequent section, we present the results of model inversion using two (stochastic and spectral) inversion schemes. We also describe the use of Bayesian model reduction procedures that are analogous to thresholding in graph theoretic analyses. The implicit induction of sparsity can be very useful for subsequent graph theoretical analysis, interpretation, and reducing the multiple comparisons problem. The final section concludes with a discussion of future applications and implications of the procedures described in this paper.

## METHODS AND MATERIALS

### Dynamic Causal Modeling for Resting-State fMRI

Resting-state fMRI is a paradigm that has become very popular during the past decade or so. This is largely because the data are easy to acquire and they disclose the intrinsic architecture of the brain in the absence of experimental or exogenous inputs. In the absence of external inputs, neuronal dynamics are driven by intrinsic activity, known as endogenous or neural fluctuations that are internal to the system. The generative models for resting-state fMRI time series have the same form as DCMs of task fMRI but discount exogenous modulatory inputs. These models can be formulated as a Taylor expansion, retaining only the linear terms; namely,

$$\dot{\text{x}}(t)=\text{A}\text{x}(t)+\text{C}\text{u}(t)+\text{v}(t)$$

$$\text{y}(t)=h\left(\text{x}(t),\text{θ}\right)+\text{e}(t).$$

The matrix **A** is known as the adjacency matrix or Jacobian describing the behavior—that is, the effective connectivity—of the system near its fixed point (*f*(***x***_***o***_) = 0), in the absence of the neuronal fluctuations **v**(*t*) and the experimental inputs**u**(*t*). In fMRI, the mapping from hidden neuronal states, **x**(*t*), to the observed blood oxygenation level dependent (BOLD) fMRI data **y**(*t*) is based on a hemodynamic model, which transforms hidden neuronal states of each population or region into predicted BOLD responses, using a previously established biophysical model (K. J. Friston et al., 2003). Here **e**(*t*) represents the measurement error and ***θ*** are parameters of the hemodynamic response function or convolution kernel *h*(**x**(*t*),***θ***).

There are currently two schemes for inverting models of resting fMRI. Although both schemes use the same variational Bayes procedures for model inversion, they differ in the data features they use for parameter estimation. The first scheme inverts data in the time domain and the model is used to predict the time series per se. This is referred to as stochastic DCM (Friston, Stephan, Li, & Daunizeau, 2010; Li et al., 2011). This requires estimation of not only the model parameters, but also the hidden states that become random variables. In terms of temporal characteristics, the hidden states are time-variant, whereas the model parameters are time-invariant. This poses a difficult inverse problem that is computationally demanding, especially when the number of hidden states (i.e., the graph) becomes too large. To finesse this problem, we proposed a DCM based upon a deterministic model (Friston, Kahan, Biswal, et al., 2014; Razi et al., 2015). This scheme provides a constrained inversion of the stochastic model by parameterizing the cross spectral density of neuronal fluctuations; namely,

$${\text{g}}_{\text{v}}\left(\omega ,\theta \right)=\alpha_{\text{v}}\omega^{-\beta_{\text{v}}}$$

$${\text{g}}_{\text{e}}\left(\omega ,\theta \right)=\alpha_{\text{e}}\omega^{-\beta_{\text{e}}}.$$

Here **g**_**x**_(*ω*) = **X** (*ω*) **X**(*ω*)^†^ represents the complex cross spectra, where **X**(*ω*) is the Fourier transform of the **x**(*t*), {**α**, **β**} ⊂ **θ** are the parameters controlling the amplitudes and exponents of the spectral density of the neural fluctuations, and *ω* = 2*πf* is the angular frequency. The implicit parameterization of endogenous fluctuations means that the states are no longer probabilistic (in contrast to stochastic DCM). This means the inversion is significantly simpler, requiring estimation of only the (time-invariant) parameters of the endogenous fluctuations and the effective connectivity. In other words, spectral DCM estimates the time-invariant parameters of models that generate observed (complex) cross spectra. In an earlier study (Razi et al., 2015), we compared and contrasted spectral and stochastic DCM with endogenous fluctuations (also known as state noise) on hidden states of models with a small number of nodes (i.e., a four-node graph). We showed that spectral DCM was not only more accurate and computationally efficient, but also more sensitive to group differences. This makes spectral DCM an ideal method for inferring effective connectivity in large brain networks. However, there is a potential to invert large-scale network models even more quickly, by calling on a previously established device that uses a prior constraint to reduce the effective number of free parameters (Seghier & Friston, 2013).

### Parameter Constraints Under Functional Connectivity Priors

As noted above, large-scale networks entail many free parameters, which make inference on large graphs computationally slow, precluding their use in large-scale studies. Furthermore, the large number of connectivity parameters can inflate model complexity, leading to potential problems with overfitting. However, it is possible to ease this problem via a simple trick; namely, by using plausible priors to constrain the number of extrinsic coupling parameters. Here, we applied the procedure detailed in Seghier and Friston (2013). In brief, this procedure involves using functional connectivity to furnish priors on effective connectivity. Although the absence of an effective connection does not preclude a functional connection (that can be mediated vicariously through other nodes), the absence of a functional connection means that the effective connection is, a priori, unlikely. This means that we can use functional connectivity to place shrinkage priors on implausible effective connections. Furthermore, one can substantiate these priors by evaluating the increase in model evidence as one increases the number of prior constraints. In practice, we do not consider each connection individually but deal with mixtures of effective connectivity that can be regarded as coupling different patterns of nodes. These patterns are referred to as *modes*. Effectively, this means that we can test the hypothesis that distributed brain responses are mediated by directed coupling among spatial patterns or modes (Friston, 2009; Seghier & Friston, 2013).

In summary, the problem of overparameterization can be finessed by replacing priors on coupling among *nodes* with priors on coupling among *modes*—where modes correspond to the principal components of the functional connectivity matrix. This provides an efficient and informed dimension reduction of the (priors over the parameters of a) large graph, based on the functional connectome. Formally—following the formulation provided in Seghier and Friston (2013)—the priors used in DCM on the exogenous connections are given by

$$p\left(A_{ij}M\right)=\text{N}\left(\mu_{ij},v_{ij}\right),$$

which leads to the diagonal form for prior covariance over extrinsic connectivity parameters:

$$\Sigma =\text{diag}\left(\text{vec}\left(v\right)\right)\in \;R^{n^{2\;}\text{x}n^{2}}.$$

Here, *n* is the number of nodes in the model. This diagonal form means that we have no prior beliefs about dependencies among various parameters. In other words, the parameters are assumed to be conditionally independent. However, if we introduce some conditional dependencies then we can decrease the effective number of parameters (i.e., rank of the prior covariance matrix). An informed way of introducing dependencies is to use the functional connectivity as a prior constraint: for example, by decomposing the BOLD signal using singular value decomposition (SVD) and then using its eigenvectors to reduce the rank of the prior covariance matrix **Σ**. Mathematically, let **Y** be the set of BOLD responses in nodes:

$$Y=\left[y_{1},y_{2},\ldots y_{n}\right]{\in R}^{n\;\text{x}t},$$

where **y**_**1**_, **y**_**2**_, … , **y**_**n**_ are the regional time series in regions 1, 2, …n. By using singular value decomposition, we can find the principal modes:

$$Y=USV^{T},$$

where $U{\in R}^{n\;\text{x}n}$ is the unitary matrix containing the modes or eigenvectors, $S{\in R}^{n\text{x}t}$ is a diagonal matrix of singular values, and $V{\in R}^{t\;\text{x}t}$ is the unitary matrix of eigenvariates. We then select the modes ***m*** with the largest singular values to remove minor modes from the prior covariance as follows:

$$\Sigma_{m}=K_{m}\Sigma K_{m}^{T},$$

where

$$K_{m}=\left[U_{m}U_{m}^{T}\right]⊗\left[U_{m}U_{m}^{T}\right].$$

Here ⊗ denotes the Kronecker product. With this formulation, we have effectively used **K**_*m*_ to introduce prior correlations so that **Σ**_***m***_ is no longer diagonal (for an illustration see Seghier & Friston, 2013). In other words, we have reduced the rank of the prior covariance matrix and the effective number of connectivity parameters. The best number of principal modes *m* can then be optimized using Bayesian model selection (or reduction), thereby providing evidential support for the hypothesis that functional connectivity provides useful priors on effective connectivity.

### Empirical Data and Model Specification

We used the open-access Oxford dataset from the FC1000 project. This dataset contains 22 adults (12 males) with a mean age of 29 years. Scanning was performed at the University of Oxford Centre for Clinical Magnetic Resonance Research using a 3-T Siemens Trio scanner with a 12-channel head coil. Whole-brain functional imaging was performed using a gradient echo EPI sequence (repetition time (TR) = 2,000 ms, echo time (TE) = 28 ms, flip angle = 89°, field of view = 224 mm, voxel dimension = 3 × 3 × 3.5 mm, acquisition time = 6 min 4 s). High-resolution anatomical 3D T1-weighted MRI scans were acquired using a magnetization-prepared rapid gradient echo sequence (TR = 2,040 ms, TE = 4.7 ms, flip angle = 8°, field of view = 192 mm, voxel dimension = 1 mm isotropic, acquisition time = 12 min). Participants were instructed to lie in dimmed light with their eyes open, think of “nothing in particular,” and not fall asleep. From the functional data containing 180 consecutive image volumes per participant, the first five volumes (dummy scans) from each participant were removed.

Data were preprocessed using standard procedures in SPM (Penny, Friston, Ashburner, Kiebel, & Nichols, 2011) as follows: data were realigned, normalized to Montreal Neurological Institute (MNI) space, and spatially smoothed using a 6 mm full width at half maximum (FWHM) Gaussian kernel. A general linear model (GLM) containing only movement (confound) regressors was constructed and inverted. An adjusted time series from the lateral ventricle was included in subsequent GLMs as an additional confound. To identify nodes, the resting state was modeled using a GLM containing a discrete cosine basis set with frequencies ranging from 0.0078 to 0.1 Hz (Fransson, 2005; Kahan et al., 2014), in addition to the aforementioned nuisance regressors. Data were high-pass filtered to remove any slow frequency drifts (< 0.0078 Hz) in the normal manner. An *F* contrast was specified across the discrete cosine transformation, producing an SPM that identified regions exhibiting BOLD fluctuations within the modeled frequencies of interest.

We used the 36 regions of interest (ROI) with coordinates from Raichle (2011) representing seven resting-state networks: default mode network, dorsal attention network, executive control network, salience network, sensorimotor system, and visual and auditory networks. The principal eigenvariate from a (8 mm radius) sphere centered on the peak *F* value from each region was computed for each region and adjusted for confounds. Table 1 gives the ROI names and their respective MNI coordinates.

**Table 1.** This table shows the 36 ROIs (names and MNI coordinates) that we adopted from Raichle (2011). The selected 36 regions belong to seven large-scale networks.

	**Region name**	**Coordinates (in mm)**		**Region name**	**Coordinates (in mm)**
**Default mode network**			**Salience network**		
1	Posterior cingulate/Precuneus	0 -52 7	23	Dorsal anterior cingulate	0 21 36
2	Medial Prefrontal	-1 54 27	24	Left anterior PFC	-35 45 30
3	Left lateral parietal	-46 -66 30	25	Right anterior PFC	32 45 30
4	Right lateral parietal	49 -63 33	26	Left insula	-41 3 6
5	Left inferior temporal	-61 -24 -9	27	Right insula	41 3 6
6	Right inferior temporal	58 -24 -9	28	Left lateral parietal	-62 -45 30
7	Medial dorsal thalamus	0 -12 9	29	Right lateral parietal	62 -45 30
8	Left posterior cerebellum	-25 -81 -33	**Sensorimotor network**		
9	Right posterior cerebellum	25 -81 -33	30	Left motor cortex	-39 -26 51	
**Dorsal attention network**			31	Right motor cortex	38 -26 48
10	Left frontal eye field	-29 -9 54	32	Supplementary motor area	0 -21 48
11	Right frontal eye field	29 -9 54	**Visual network**		
12	Left posterior IPS	-26 -66 48	33	Left V1	-7 83 2
13	Right posterior IPS	26 -66 48	34	Right V1	7 83 2
14	Left anterior IPS	-44 -39 45	**Auditory network**		
15	Right anterior IPS	41 -39 45	35	Left A1	-62 -30 12
16	Left MT	-50 -66 -6	36	Right A1	59 -27 15
17	Right MT	53 -63 -6			
**Control executive network**					
18	Dorsal medial PFC	0 24 46			
19	Left anterior PFC	-44 45 0			
20	Right anterior PFC	44 45 0			
21	Left superior parietal	-50 -51 45			
22	Right superior parietal	50 -51 45			

### DCM Specification, Inversion, and Reduction

We proceeded to specify a fully connected 36-node DCM, without exogenous inputs, for each of the 22 participants separately. We inverted these specified DCMs using both stochastic and spectral DCM. For 3 participants stochastic DCM failed to converge after 128 iterations, so we discarded those participants from subsequent analysis, yielding a set of 38 inverted DCMs altogether (i.e., 2 DCM schemes × 19 subjects).

In terms of computational time, a graph with 36 nodes takes about 200 min per iteration for stochastic DCM and around 20 min per iteration for spectral DCM with convergence achieved within 64–128 iterations. This means that spectral DCM is considerably more efficient than stochastic DCM (around 10 times faster). It is also more robust as it successfully inverted all models, whereas stochastic DCM failed to invert 3 DCMs (see above).

We then used Bayesian model reduction (Seghier & Friston, 2013) to optimize the number of prior modes in the DCMs, based on the modes (i.e., eigenvectors) of the functional connectivity matrix as described above. Bayesian model reduction enables the experimenter to analytically derive the free energy (as a proxy for log-model evidence) and parameters for inverted DCM with modified prior covariance matrices. We therefore inverted a fully connected DCM (using spectral and stochastic schemes) for each subject, without any constraints on the prior covariance matrix. We then used Bayesian model reduction to calculate the free energy for variants of the DCM with a different number of prior modes. This means that the constraints on the prior covariance are data driven (see definition of **K**_*m*_ and **Σ**_***m***_ above) and varied from subject to subject. In routine analyses, we envisage that the initial (fully connected) inversion would start with a relatively small number of modes (e.g., *m* = 10: see below), which reduces computation time considerably.

## RESULTS

**Figure 1.**
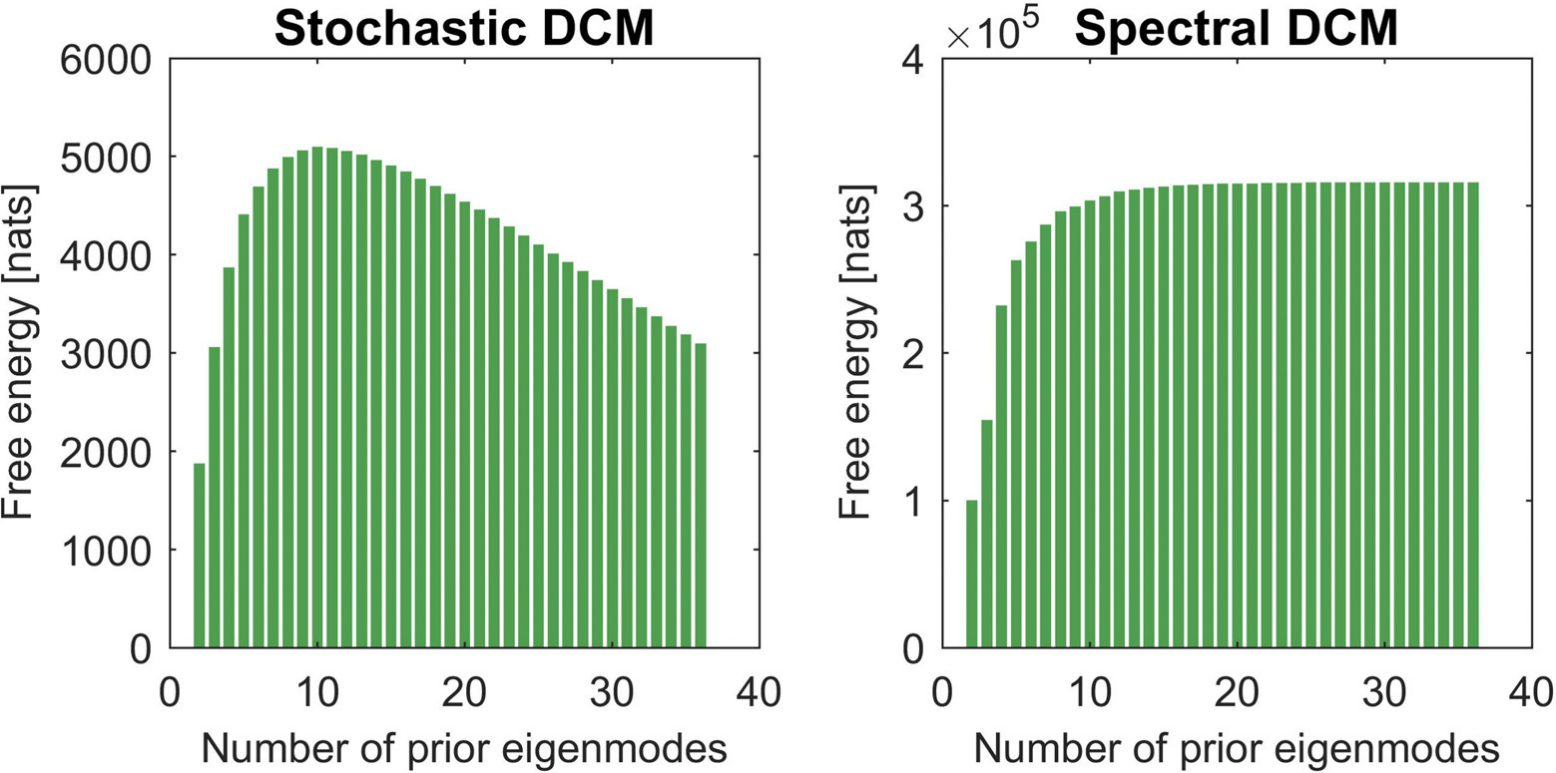
This figure shows the plots of the averaged free energy over participants as we increase the number of prior eigenmodes *m*. The left panel shows the profile of free energy (as a proxy for log-model evidence) for stochastic DCM. One can see that free energy first increases with a peak at *m* = 10 and then decreases. Using 36 modes (equal to the number of nodes) means that there are effectively no prior constraints. In the right panel, we show a similar plot for spectral DCM. We now see that the free energy plateaus at around *m* = 10.

Effective connectivity was computed using both inversion schemes; namely, stochastic and spectral DCM. Figure 1 (left panel) shows the average free energy (log evidence), in nats, over 19 participants over the number of modes *m* relative to the free energy at *m* = 1 using stochastic DCM. The plot shows that free energy first increases and then systematically decreases with increasing number of modes (peak at *m* = 10). The right panel shows results when the inversion was performed using spectral DCM. We again see a similar trend, but this time the free energy reached a plateau at around *m* = 10 modes. Thus, in contrast to stochastic DCM, the uncertainty about the exact number of modes is not a critical issue for spectral DCM, because any intermediate number of modes (within the plateau in Figure 1) would yield the same (high) model evidence. In summary, this analysis shows that by replacing connections between many *nodes* with connections between a small number of *modes*(here *m* = 10), effective connectivity of large graphical models can be optimally estimated with both spectral and stochastic DCM.

**Figure 2.**
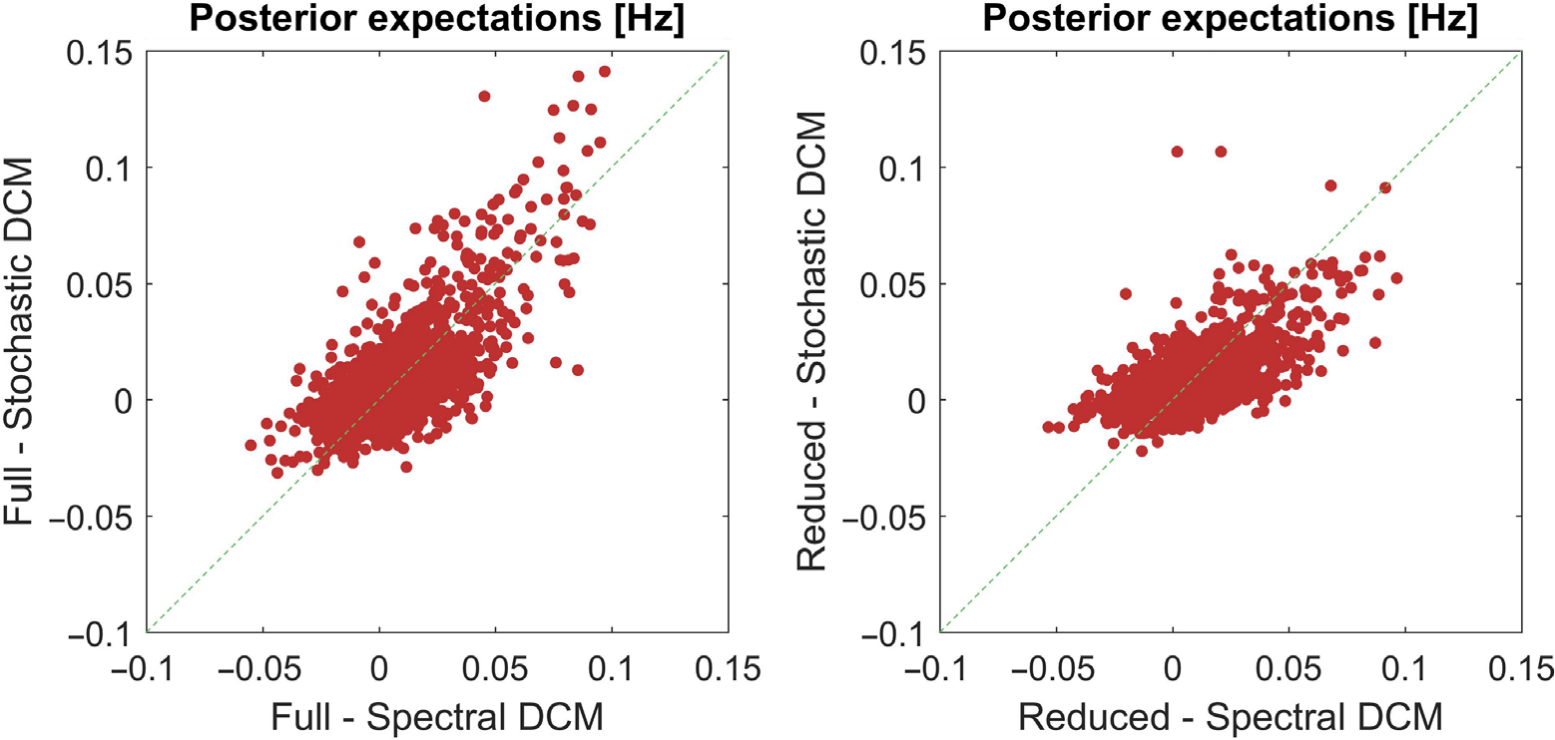
This figure uses regression plots to illustrate the correspondence between the averaged parameter estimates over participants from two (stochastic and spectral) inversion schemes. The left panel shows the relationship in the absence of any prior constraint. We see that the parameter estimates are highly correlated (rho = 0.67). The plot on the right shows the equivalent results when we used an optimal number of prior modes, in terms of those that maximizes the free energy, for each subject and then averaged the parameter estimates over participants. We again see that there is high correlation between the parameter estimates of the two inversion schemes (rho = 0.65). We excluded the self-connections (diagonal entries) when doing this analysis as they are scaled parameters.

Figure 2 presents regression plots that compare the posterior estimates (i.e., expectations of effective connectivity parameters) from the two inversion schemes. The left panel shows the parameter estimates, averaged over participants, from the two inversion schemes when all modes were used; that is, no post hoc model reduction is employed and the number of modes was equal to the number of nodes (*m* = *n* = 36). Here, we see the parameter estimates from the two schemes are highly correlated (rho = 0.67). The right panel shows the regression plot when we used the optimal number of modes for each subject for both inversion schemes (*m* = 10). The two inversion schemes still return highly correlated parameters (rho = 0.65).

**Figure 3.**
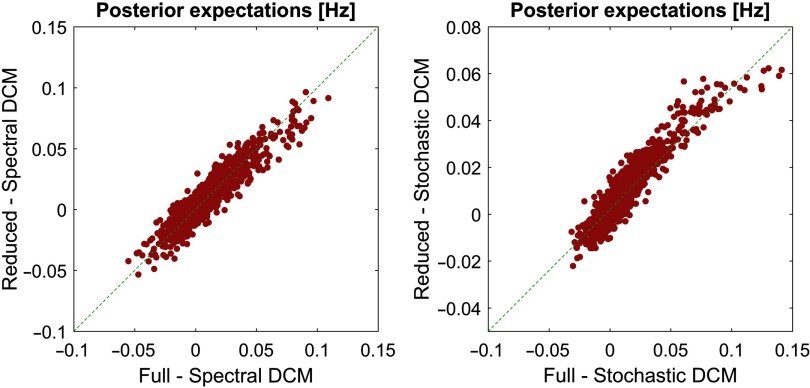
This figure shows the regressions comparing the parameter estimates for each inversion scheme separately. On the left, we compare the averaged parameter estimate from spectral DCM when we used no prior constraint (full) and when we used an optimal number of modes, selected on the basis of free energy, for each subject (reduced). We see that the parameter estimates are highly correlated (rho = 0.93). The right-hand plot shows the parameter estimates for stochastic DCM, which also evidence high correlations (rho = 0.94).

In Figure 3 we again show the regression plots, but this time we were interested in comparing the validity of parameter estimates for each scheme separately. The left panel shows the spectral DCM results, when we plot the parameter estimates, averaged over participants, when no reduction was performed against the parameter estimates when using the optimal number of modes for each subject. This reveals a very high conformance between the parameter estimates (rho = 0.93). The right panel shows the equivalent plot for stochastic DCM. We again see highly correlated parameter estimates (rho = 0.94).

**Figure 4.**
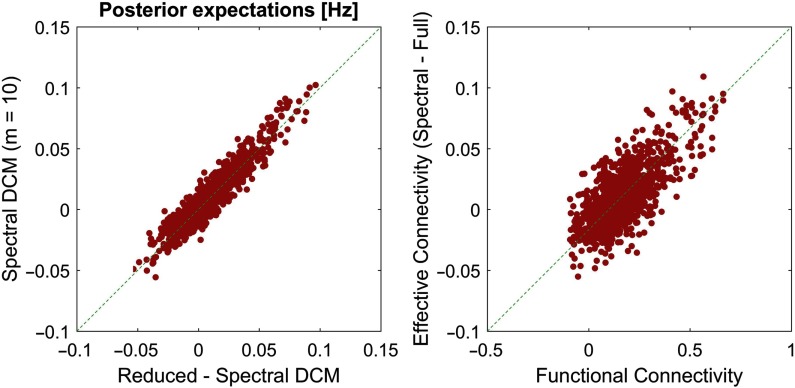
This figure shows the regression of parameter estimates from spectral DCM (left panel) and the high conformance between functional and effective connectivity (spectral DCM with all modes). The plot in the left panel illustrates the validity of parameter estimates when we used *m* = 10 modes for every participant, relative to using an optimal number of modes for each subject. We again see a high correlation between the estimated connectivity parameters (rho = 0.94). The right panel plots functional connectivity against effective connectivity, which unsurprisingly showed a strong correlation (rho = 0.70).

Figure 4 uses the same format but this time only for the spectral DCM (left panel). Here, we were interested in the consistency of parameter estimates when fixing ***m*** to 10 modes for all subjects, against using the optimal number of modes for each subject. Pleasingly, we see that there was very high correlation between the parameter estimates (rho = 0.94). For completeness, we also plot (on the right panel) the high conformance between effective (using spectral DCM) and functional connectivity (rho = 0.70), which is not surprising as the effective connectivity are the causes that engender the observations or the functional connectivity. The results shown in Figures 3 and 4 demonstrate the construct validity of stochastic and spectral DCM. Furthermore, they show that the functional connectivity priors have empirical validity. This is because their application increases model evidence—and that the number of linear prior constraints (functional connectivity modes) is roughly the same for spectral and stochastic models. Finally, the underlying posterior estimates of effective connectivity do not depend sensitively on the number of prior constraints. In the next analyses, we used Bayesian model reduction to examine the contribution of individual connections to model evidence, as opposed to modes or patterns of connections illustrated above. This application of Bayesian model reduction to individual connections aims to discover the structure of the optimal sparse graph.

**Figure 5.**
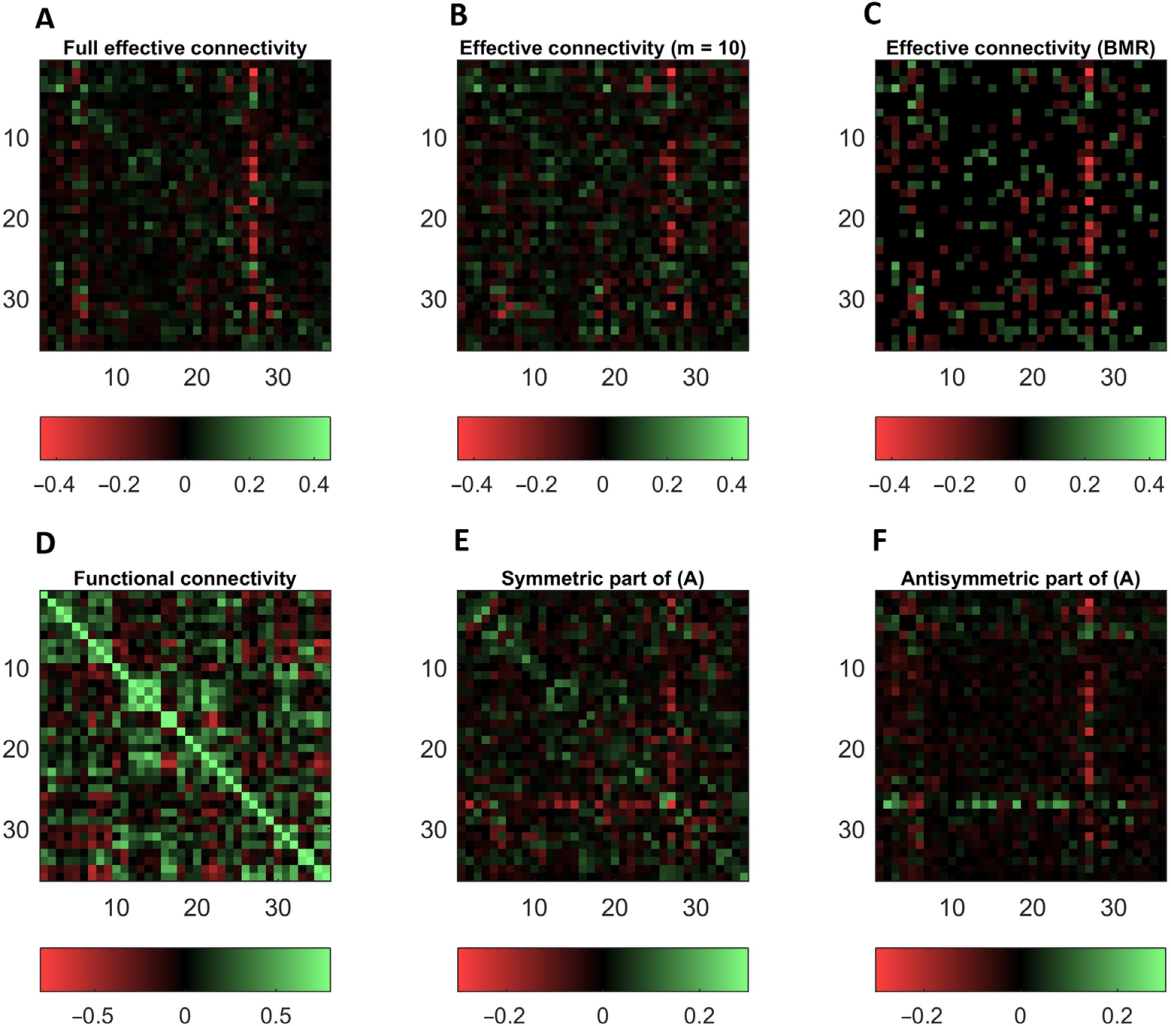
This figure illustrates the sparse structure of effective connectivity after applying Bayesian model reduction to eliminate redundant connections. Top row: These three effective connectivity matrices correspond to the full or parent estimate for this particular subject (A), the equivalent matrix following Bayesian model reduction with 10 prior modes (B), and, finally (on the right) after eliminating redundant connections (shown in dark black, which are the majority of the connections here) with Bayesian model reduction (C). Lower row: These show different characterizations of symmetric and asymmetric connectivity. The left panel (D) shows the functional connectivity matrix associated with (or generated by) the (reduced) effective connectivity on the upper right. The effective connectivity has been separated into symmetric (E) and antisymmetric components (F), in the lower middle and right panels respectively. Note the sparse nature of effective connectivity, in relation to functional connectivity (when comparing the lower left and middle panels). This difference illustrates the general phenomena that functional connections can be mediated vicariously via indirect effective connections.

Figure 5 (A, upper left panel) shows the posterior expectations, for a typical subject, after Bayesian model reduction (Friston, Li, Daunizeau, & Stephan, 2011; Friston et al., 2016) was applied to each connection in turn. We used two-tone color map throughout with excitatory connections shown as green (go) and inhibitory as red (stop). This involves comparing models with and without each connection in terms of their (reduced) free energies or model evidence. Bayesian model reduction eliminates redundant connections (shown as (dark) black on panel C), when the evidence for the sparser model exceeds that of the model that retains each connection. This represents a principled way of thresholding or eliminating connections that are not necessary to explain the fMRI data. Heuristically, redundant connections are parameters whose complexity cost exceeds the increase in accuracy or goodness of fit (noting that model evidence is equal to accuracy minus complexity). Figure 5 also shows the effect of Bayesian model reduction at the level of functional connectivity priors (upper middle panel) and at the level of individual connections (upper right panel). Crucially, the resulting extrinsic connectivity (adjacency) matrix is very sparse compared with the fully connected architecture that constitutes the parent model.

The lower panels in Figure 5 show different characterizations of connectivity. The left panel (D) shows the functional connectivity matrix associated with (or generated by) the (reduced) effective connectivity on the upper right. Note the sparse nature of effective connectivity, in relation to functional connectivity (when comparing the lower left and middle panels). This difference illustrates the general phenomena that functional connections can be mediated vicariously via indirect effective connections. The remaining panels in Figure 5 highlight the asymmetry of effective connectivity by showing the symmetric part (E, lower middle panel) and antisymmetric part (F, lower right panel). These are obtained simply by transposing the adjacency matrices and taking the sum and difference respectively (see discussion for the importance of this decomposition).

**Figure 6.**
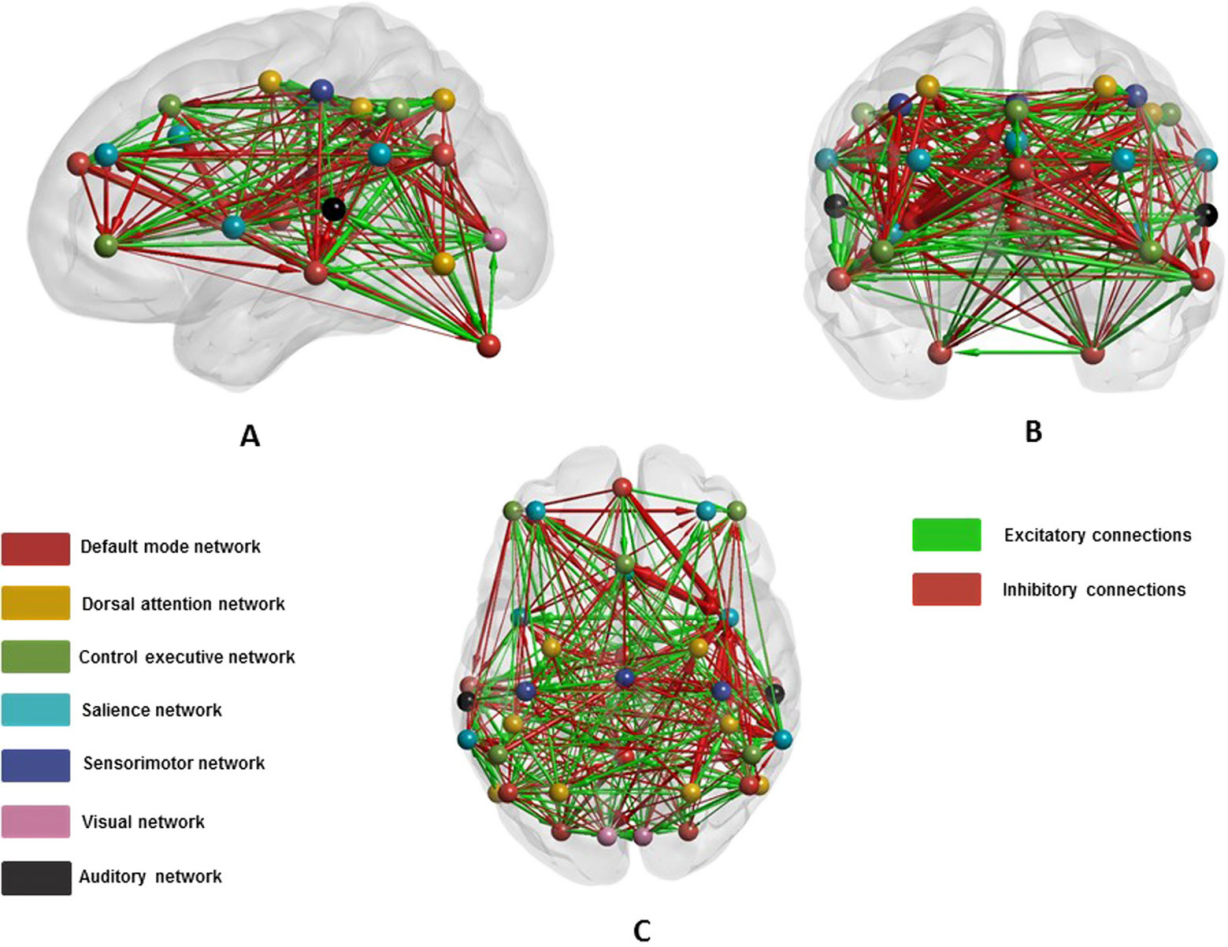
This figure shows the 36 ROIs (Raichle, 2011) that form seven large-scale brain modes or intrinsic networks. The graphics show a typical participants (same as in Figure 5) inverted graph after applying Bayesian model reduction to connections. The brain regions, represented as balls, are color-coded for various networks. The edges or connections are shown by directed arrows where the width of the arrows reflects the strength of the coupling. The color of the arrows represents the excitatory (green) and inhibitory (red) coupling among neuronal populations. We show the brain in sagittal (A), coronal (B), and axial (C) views.

In summary, this application of Bayesian model reduction (BMR) finds the best model structure by removing the edges from the large (parent) graph by comparing models with and without each connection. The resulting BMR is particularly useful for large graphs and serves to prune connections and reveal any underlying sparsity. This BMR form of model selection offers a principled alternative to the arbitrary thresholding strategies common in the graph theoretic literature. Figure 6 shows one such subject’s (the same subject as in Figure 5) graph. Note that the edges on this graphic are directed and signed, where green arrows denote excitatory (positive) connections and red arrows are inhibitory (negative) connections. The width of each connection represents coupling strength (in hertz). We have suppressed self or recurrent connections, which are—by construction—inhibitory in DCM (to model intrinsic inhibition that precludes runaway excitation).

**Figure 7.**
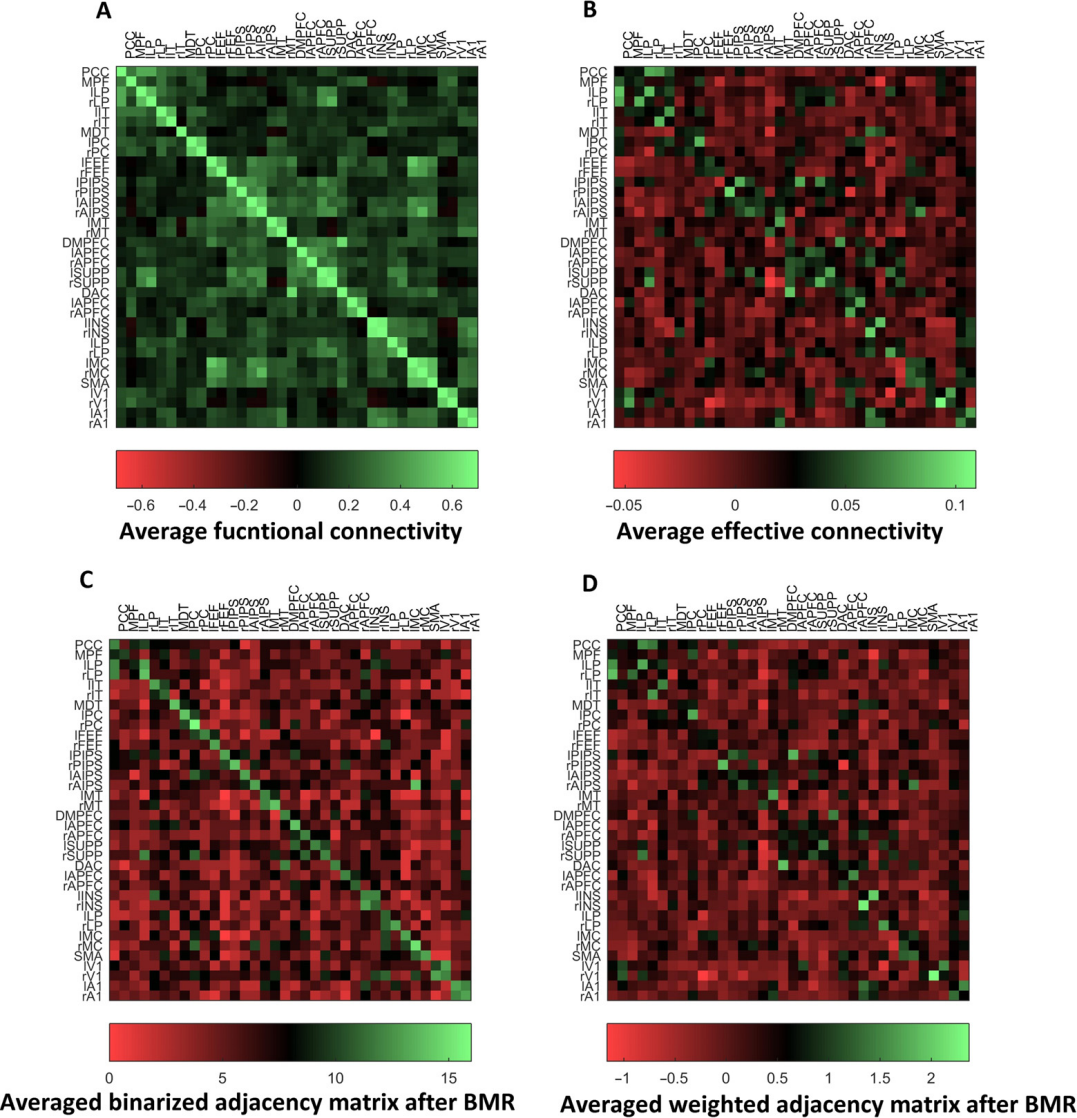
This figure shows the averaged functional (A) and effective connectivity (B) over 19 subjects. The diagonal for the functional connectivity represents the correlation of each region with itself. The correlations within each network are quite distinctive, and the relationship between networks is visually evident. We see similar patterns in the effective connectivity matrix but there are clear asymmetries in the connectivity. We have also shown averaged effective connectivity matrix after Bayesian reduction and binarization (C) and when the weights are retained (D).

Figure 7 shows the averaged functional (A) and effective connectivity (B) over subjects. The diagonal terms in the effective connectivity matrix show self-connections that are modeled as inhibitory connections. It is interesting to compare the functional connectivity with that reported in Raichle (2011) for a single subject (but for a much longer time series of around 30 min). Different modes or networks are relatively easy to identify visually as they are grouped in terms of functional connectivity. This structure is less obvious with effective connectivity, which is asymmetric. Note that functional connectivity is based on sampled time series and is therefore confounded by the observation (or thermal) noise. We have also plotted averaged effective connectivity after removing redundant connections with Bayesian model reduction and binarizing the effective connectivity (i.e., setting the adjacency matrix to one if the connection exists for each subject) (C); the ensuing average reflects the number of times a connection is evident over subjects. Finally, we present the associated (averaged effective connectivity) matrix after Bayesian model reduction (D) for comparison with (B).

**Figure 8.**
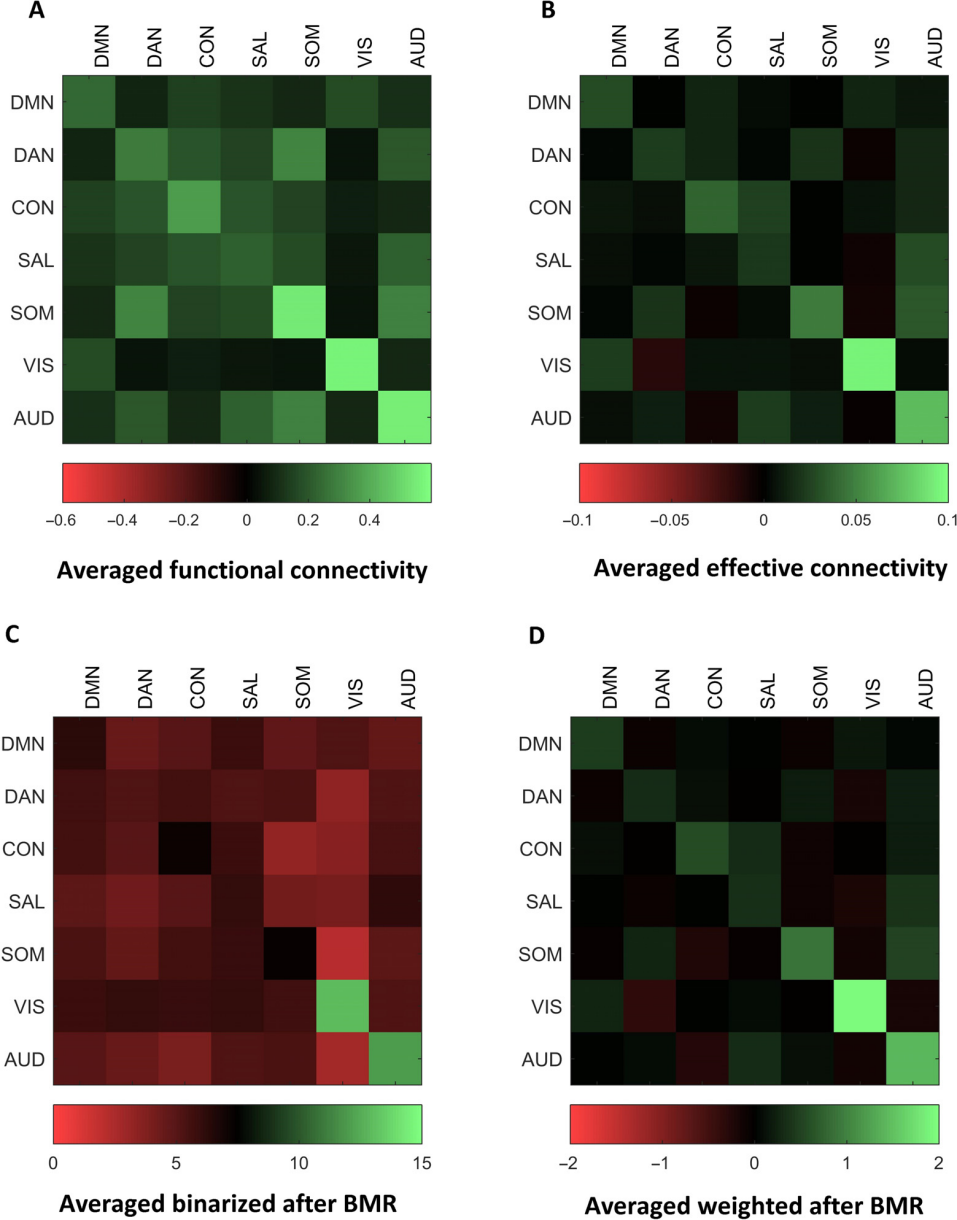
This figure is in the same format as Figure 7. The graphics show the averaged functional (A) and effective connectivity (B) over 19 subjects after down-sampling the 36 ROIs to the seven networks or *modes*. For the seven modes, we have also plotted averaged effective connectivity matrix after Bayesian reduction and binarization (C) and when the weights are retained (D).

To illustrate how different brain networks or *modes* are functionally integrated, Figure 8 shows the connectivity matrices from Figure 7 but after down-sampling to connect the constituent modes. This down-sampling entails averaging effective connectivity strengths among the sets of regions that constitute each node. It is interesting to note that the functional connectivity, as plotted in (A), between modes is exclusively positive whereas there are both excitatory (positive) and inhibitory (negative) influences among different modes in the effective connectivity matrix (B). These are even more pronounced in the averaged connectivity matrix after Bayesian model reduction (D).

**Figure 9.**
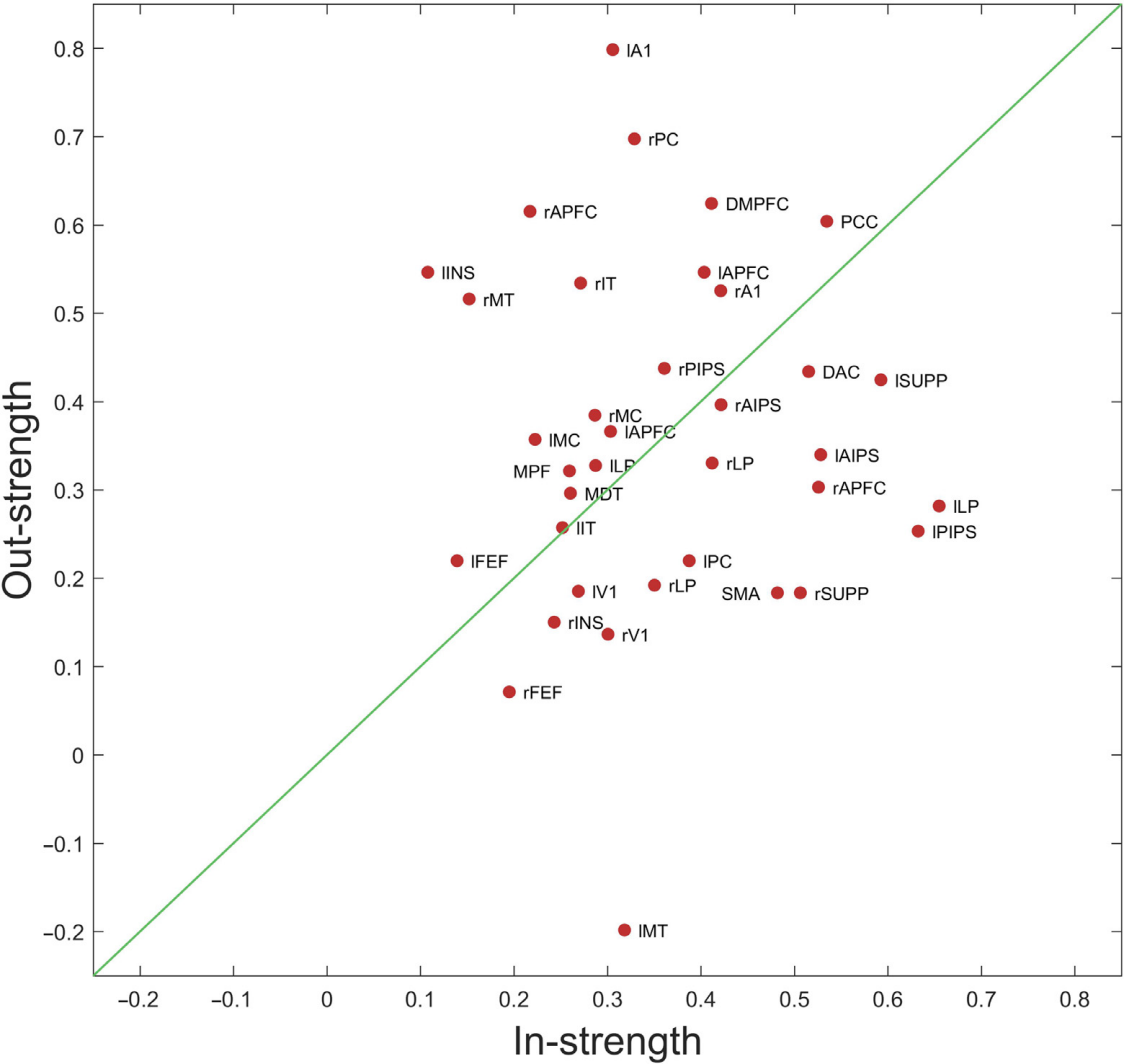
This figure shows the hemispheric asymmetries as a scatter plot, using the averaged effective connectivity estimates as shown in Figure 7B. We used nodal in-strengths and out-strengths to identify these asymmetries. The in-strength summarizes the sum of all weighted connections entering the node, while the out-strength is the sum of all the weighted connections going out from a particular node. On the scatter plot, regions that lie above the diagonal line are net senders or sources, whereas regions that lie below the diagonal line are the net receivers or sinks.

**Figure 10.**
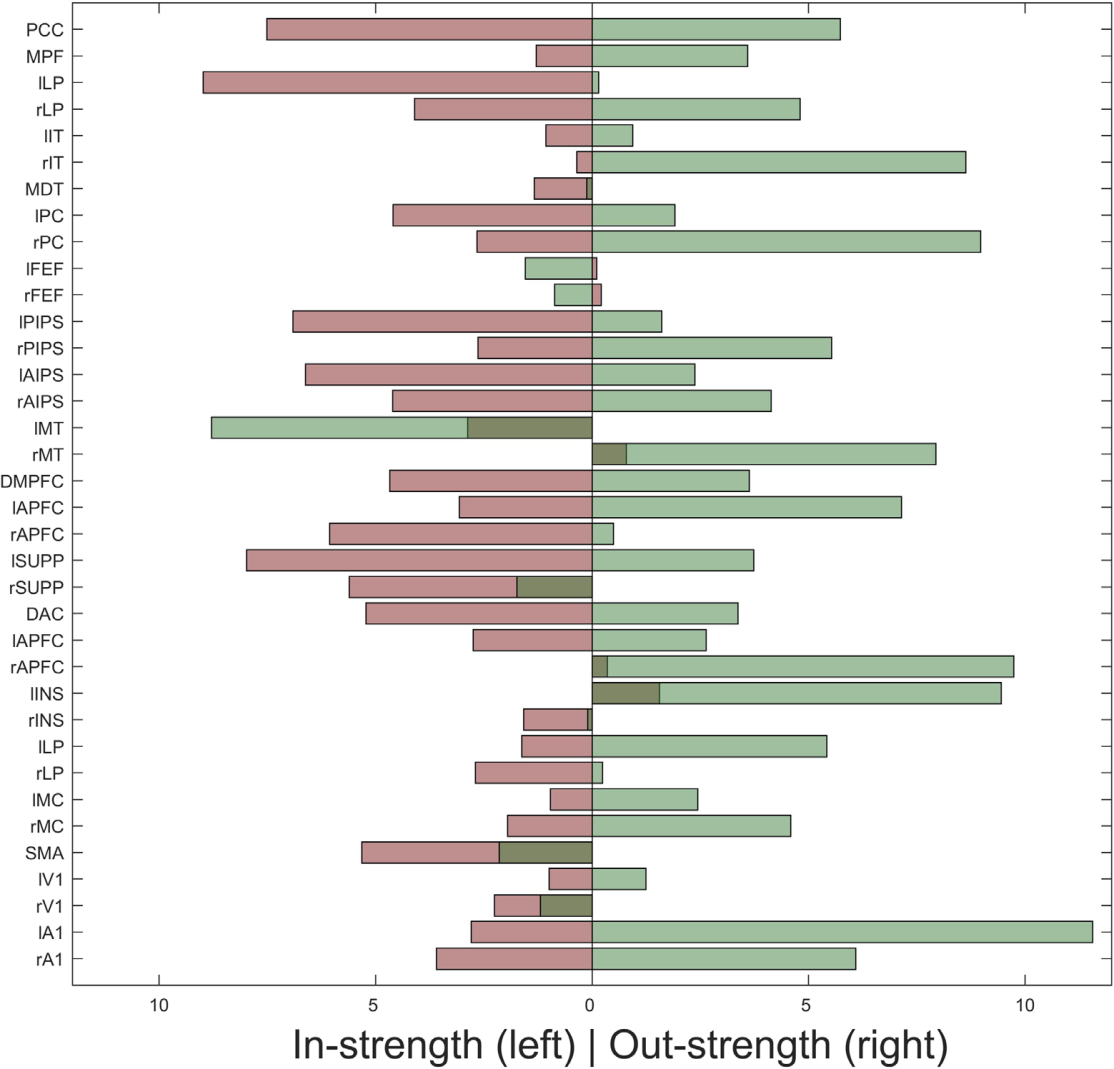
This figure shows the hemispheric asymmetries as a bar plot based on the effective connectivity estimates after Bayesian model reduction as shown in Figure 7D. The nodal in-strength and out-strength are calculated as in Figure 9.

In our final analysis, we looked more closely at asymmetric nature of the effective connectivity afforded by the spectral DCM. These asymmetries are fundamental to characterize the organization of the cortex—in terms of hierarchies or lateralization—that cannot be disclosed with undirected measures of connectivity. As an example, we examine the hemispheric asymmetries in Figure 9, which shows these asymmetries as scatter plot using the averaged effective connectivity matrix from Figure 7B. The scatter plot shows the nodal in-strength versus out-strength such that the regions that lie above the line are net senders or sources, that is, they have greater out than in-strength, whereas the regions that are below the diagonal line are the net receivers or sinks. There are very notable hemispheric asymmetries; for example, notice the difference between left and right fontal eye field (FEF) or left and right insula (INS). The hemispheric asymmetries are even clearer when we used the averaged effective connectivity matrix in Figure 10, after Bayesian model reduction, as shown in Figure 7D, using a bar plot. The bar plot shows the nodal in-strength (light red bars) and out-strength (light green bars). There are notable hemispheric asymmetries: for example, the difference between left and right lateral parietal lobules or left and right insula (INS). These are interesting observations attesting the utility of spectral DCM for characterizing large-scale brain networks at a level not accessible previously. It is important to remember that these results are based upon one dataset and are just used to illustrate the sorts of analyses that can be performed.

## DISCUSSION

In this paper, we have described a framework for estimating effective connectivity from fMRI data collected at rest. Our framework builds upon three recent developments: (a) a robust and fast inversion scheme called spectral DCM (Friston, Kahan, Biswal, et al., 2014), (b) an informed data-driven procedure to reduce the effective number of parameters in large DCMs (Seghier & Friston, 2013), and (c) a principled network discovery procedure that produces sparse graphs using Bayesian model reduction (Friston et al., 2016). We have demonstrated the construct validity of this framework using empirical fMRI data with large DCMs (36-node graphs).

Specifically, we have shown that one can use spectral DCM to infer large-scale networks describing whole-brain connectivity efficiently from resting-state fMRI. We have demonstrated that the connectivity estimates from two (stochastic and spectral) inversion schemes for resting-state fMRI are internally consistent, in terms of high correlations, when averaged over participants. However, spectral DCM is computationally much more efficient (approximately 10 times faster in this study) by virtue of using a deterministic generative model of spectral data, obtained after transformation of time series to the spectral domain (Friston, Kahan, Biswal, et al., 2014). This spectral formulation eludes the computational burden of estimating hidden states per se, hence speeding up model inversion. It is noteworthy that the inversion of spectral DCM is even faster than conventional deterministic DCM, since it does not require the integration of differential equations.

In principle, there are no limits on the size of the graph, given sufficient memory and computational power. However, there are practical limitations: inverting a large model with many parameters can entail slow convergence—taking around 64 to 128 iterations—where each iteration takes longer as the graph size increases. The bottleneck here becomes memory size, which may require use of high-performance computing facilities (that are available at some institutions). Practically speaking, we have inverted graphs as large as 64 nodes. One can invert larger graphs; for example, by splitting the graphs into two (e.g., for each hemisphere). One can then use the posterior estimates of subgraphs as the initial values for inverting a full graph, that then converge more quickly.

There are several advantages such large graphs bring to the table. First, they are inherently directed. Here, causality is defined in control theoretic terms. In other words, causality is embedded in the generative model via differential equations that model interactions and evolution of latent neural states that cause the measured BOLD responses. This is in contrast to directed measures of functional connectivity; for example, Granger causal modeling that is based on temporal precedence (Friston, Moran, & Seth, 2013; Razi & Friston, 2016). Second, the edges or connections of these graphs are weighted, representing the coupling strengths between regions. Third, they are signed, where the positive and negative edges model excitatory and inhibitory influences on neuronal populations. The potential asymmetry between reciprocal connections is of fundamental importance for brain connectivity. For example, neurobiological formulations of cortical hierarchies in terms of feedforward (usually excitatory) and feedback (usually inhibitory) connections rest on asymmetric directed connectivity. Finally, the diagonal entries on the adjacency matrix are also weighted, representing the self-connections or activity within region that is modeled as self or recurrent inhibition.

The proposed framework can be usefully employed to address some of the methodological challenges faced by the (functional) connectomics. We note one of the limitations of graphs based on functional (and structural) connectivity; while some techniques use the full connectivity matrix, such as for community detection (Rubinov & Sporns, 2011), most extant network studies have employed thresholding to remove spurious connections and to suppress negative correlations in functional networks (hence functional connectomes are usually unsigned). There are several ways to threshold (i.e., absolute, proportional, or based on group thresholding methods) and little consensus on the best way to threshold (Qi, Meesters, Nicolay, Romeny & Ossenblok, 2015; Simpson, Bowman, & Laurienti, 2013). Furthermore, thresholding removes many (functional connectivity) data features. This is potentially important because the metrics calculated from the ensuing sparse graphs are sensitive to the amount and the method of thresholding (usually requiring the computation of graph theoretic descriptors over several thresholding levels). The Bayesian framework used by DCM allows for an informed and graceful way of performing “network inference” via Bayesian model reduction to select or threshold out redundant edges (Friston et al., 2016). This model reduction procedure, now in wide use, allows one to infer the best model that is nested within the parent or fully connected model. In summary, Bayesian model reduction can be used to disclose the underlying sparsity by comparing the evidence for models with and without particular connections, and thus enables a principled way to perform graph-based functional network analysis.

Another interesting issue in this field is the availability of tools to analyze directed graphs. Although there has been a huge interest and increasing sophistication in the use of graph theoretic summary measures (for example clustering coefficient, path length, efficiency, and modularity), most of the existing literature (at least within human neuroimaging) is based on undirected and unsigned anatomical and functional connectivity. However, as empirical work in animal models amply demonstrates, anatomical neural projections and their physiological interactions are fundamentally directed and weighted, rendering binary and undirected graphs relatively poor approximations. One interesting aspect of directed graphs or adjacency matrices is that they cannot be embedded in metric spaces. For example, several procedures (e.g., multidimensional scaling and spectral embedding) are based upon the eigenvectors of the adjacency matrix that assume the adjacency matrix is symmetric. Put simply, this means that the coupling between two nodes can be treated as a distance in some metric space, thereby affording the opportunity to understand the relationship between nodes in terms of distances between them. This facility disappears in the context of weighted and directed graphs. For example, it is impossible to have a negative distance in signed graphs. A more subtle point is that weighted graphs mean that node A can be close (or similar) to node B, while node B can be distant (or dissimilar) from node A. So what are the emerging tools for characterizing weighted graphs in functional connectomics?

Spectral graph theory theory has not attracted much attention in neuroimaging but may be an emerging candidate. For example, Raj, Kuceyeski, & Weiner (2012) use spectral graph theory through the properties of the graph Laplacian and its eigenspectrum. Spectral graph theory can be a useful tool because the Laplacian—and its eigenspectrum—contains all the information necessary to characterize a graph. Developing efficient algorithms for finding community structure and modules via the Laplacian of directed (hyper)graphs is still a nascent field (Chung, 2005). We have previously proposed a variant of DCM (Friston, Kahan, Razi, Stephan, & Sporns, 2014) using a generative model that used the eigenmodes and associated eigenspectrum to parameterize the effective connectivity. However, this formulation rests upon a symmetry constraint—and therefore deals with real eigenvalues—to provide estimates of (symmetric) connectivity and the underlying topology, where the connectivity between nodes depends upon their location in a multidimensional scaling space. Although this approach is mathematically elegant, it would be nice to have equivalent procedures for directed graphs whose Laplacian has complex eigenvalues (with imaginary parts). It is interesting to note that state-of-the-art functional connectivity studies appeal to the notion of hierarchies in understanding principal modes of functional connectivity (e.g., Margulies et al., 2016), despite the fact that the asymmetries in connectivity that define hierarchies are precluded in undirected (functional connectivity) graphs.

Although convenient, the symmetry of undirected graphs compromises their biological plausibility by denying asymmetries; for example, in terms of feedforward (e.g., targeting excit atory spiny stellate neurons) and feedback (e.g., targeting inhibitory interneurons) connections in the brain. With this limitation in mind, it may be possible to relax the symmetry constraint by parameterizing a DCM with complex eigenvalues by splitting (effective) connectivity into symmetric and antisymmetric components (see the lower row of Figure 6; see also Carlson, 1999; Chung, 2005; Donetti, Neri, & Munoz, 2006). We hope to explore these avenues in future work with the ultimate aim of characterizing network architectures in clinical populations.

## ACKNOWLEDGMENTS

We would like to thank Ric Davis for providing extensive computational resources for the inversion of large stochastic DCMs.

## SUPPORTING INFORMATION

The two DCM schemes described in this paper are implemented in MATLAB code and are available freely as part of the open-source software package SPM12 (Penny et al., 2011) and its website (http://www.fil.ion.ucl.ac.uk/spm). The raw data used in this paper are openly acces sible and can be downloaded from the 1000 Functional Connectomes Project (Biswal et al., 2010) and its website (http://fcon_1000.projects.nitrc.org/fcpClassic/FcpTable.html).

## AUTHOR CONTRIBUTIONS

Adeel Razi: Conceptualization; Data curation; Formal analysis; Methodology; Visualization; Writing – original draft. Mohamed Seghier: Conceptualization: Supporting; Methodology: Supporting; Writing – review & editing: Supporting. Yuan Zhou: Contributor Information: Writing – review & editing: Supporting. Peter McColgan: Writing – review & editing: Supporting. Peter Zeidman: Writing – review & editing: Supporting. Hae-Jeong Park: Writing – review & editing: Supporting. Olaf Sporns: Visualization: Supporting; Writing – review & editing: Supporting. Geraint Rees: Conceptualization: Equal; Writing – review & editing: Supporting. Karl Friston: Conceptualization: Equal; Formal analysis: Supporting; Methodology: Supporting; Writing – original draft: Supporting; Writing – review & editing: Supporting.

## TECHNICAL TERMS

Dynamic causal modeling: A Bayesian framework that is used to infer causal interaction between coupled or distributed neuronal systems.
Effective connectivity: A measure of the directed (causal) influence of one neural system over another using a model of neuronal interactions.
Functional connectivity: A (undirected) measure of the statistical dependencies between spatially remote neurophysiological events.
Generative model: A model for randomly generating observable data values, typically given some hidden parameters.
Bayesian model selection: Procedure to determine the most likely among a set of competing hypotheses (or models) about mechanisms that generated observed data.
Bayesian model reduction: A Bayesian inversion and comparison of models that are reduced (or sparsed) forms of a full (or parent) model.
Thresholding: A procedure applied to adjacency matrix of a graph to induce sparsity by removing connections above or below a certain value.
Neural fluctuations: Refers to the random intrinsic (or spontaneous) fluctuations within neuronal networks.
Adjacency matrix: Square matrix representation of graph that is either binary (presence or absence of connections) or weighted (showing strength of connections).
Cross spectral density: A frequency domain transformation of the cross-correlation or cross-covariance between two time series signals.
Singular value decomposition: A procedure in linear algebra that factorizes a matrix into simpler and meaningful pieces.
Free energy: A lower bound on model evidence typically used for model selection; higher free energy indicates better fit of data.
Spectral graph theory: A study of the relationship between a graph and the eigenvalues and eigenvectors of its Laplacian matrix.
Graph Laplacian: A matrix representation of graph that combines node adjacency and node degree in mathematical formulation and belongs to spectral graph theory.

## References

[bib1] Biswal B. B., Mennes M., Zuo X.-N., Gohel S., Kelly C., Smith S. M., … Milham M. P. (2010). Toward discovery science of human brain function. Proceedings of the National Academy of Sciences, 107, 4734–4739. 10.1073/pnas.0911855107 PMC2842060 20176931

[bib2] Breakspear M. (2004). “Dynamic” connectivity in neural systems: Theoretical and empirical considerations. Neuroinformatics, 2(2), 205–226. 10.1385/NI:2:2:205 15319517

[bib3] Bullmore E., & Sporns O. (2009). Complex brain networks: Graph theoretical analysis of structural and functional systems. Nat Rev Neurosci, 10(3), 186–198. 10.1038/nrn2575 19190637

[bib4] Carlson R. (1999). Inverse eigenvalue problems on directed graphs. Transactions of the American Mathematical Society, 351(10), 4069–4088. 10.1090/s0002-9947-99-02175-3

[bib5] Chung F. (2005). Laplacians and the Cheeger inequality for directed graphs. Annals of Combinatorics, 9(1), 1–19. 10.1007/s00026-005-0237-z

[bib6] Donetti L., Neri F., & Munoz M. A. (2006). Optimal network topologies: Expanders, cages, Ramanujan graphs entangled networks and all that. Journal of Statistical Mechanics. 10.1088/1742-5468/2006/08/p08007

[bib7] Fornito A., Zalesky A., & Breakspear M. (2015). The connectomics of brain disorders. Nature Reviews Neuroscience, 16(3), 159–172. 10.1038/nrn3901 25697159

[bib8] Fransson P. (2005). Spontaneous low-frequency BOLD signal fluctuations: An fMRI investigation of the resting-state default mode of brain function hypothesis. Human Brain Mapping, 26(1), 15–29. 10.1002/hbm.20113 15852468 PMC6871700

[bib9] Friston K., Moran R., & Seth A. K. (2013). Analysing connectivity with Granger causality and dynamic causal modelling. Current Opinion in Neurobiology, 23(2), 172–178. 10.1016/j.conb.2012.11.010 23265964 PMC3925802

[bib10] Friston K. J. (2009). Modalities, modes, and models in functional neuroimaging. Science, 326(5951), 399–403. 10.1126/science.1174521 19833961

[bib11] Friston K. J., Frith C. D., Liddle P. F., & Frackowiak R. S. (1993). Functional connectivity: The principal-component analysis of large (PET) data sets. Journal of Cerebral Blood Flow and Metabolism, 13(1), 5–14. 10.1038/jcbfm.1993.4 8417010

[bib12] Friston K. J., Harrison L., & Penny W. (2003). Dynamic causal modelling. Neuroimage, 19(4), 1273–1302. 12948688 10.1016/s1053-8119(03)00202-7

[bib32] Friston K. J., Kahan J., Biswal B., & Razi A. (2014). A DCM for resting state fMRI. NeuroImage, 94, 396–407. 10.1016/j.neuroimage.2013.12.009 24345387 PMC4073651

[bib13] Friston K. J., Kahan J., Razi A., Stephan K. E., & Sporns O. (2014). On nodes and modes in resting state fMRI. NeuroImage. 10.1016/j.neuroimage.2014.05.056 PMC4121089 24862075

[bib14] Friston K. J., Li B., Daunizeau J., & Stephan K. E. (2011). Network discovery with DCM. NeuroImage, 56(3), 1202–1221. 10.1016/j.neuroimage.2010.12.039 21182971 PMC3094760

[bib15] Friston K. J., Litvak V., Oswal A., Razi A., Stephan K. E., van Wijk B. C. M., … Zeidman P. (2016). Bayesian model reduction and empirical Bayes for group (DCM) studies. Neuro Image, 128, 413–431. 10.1016/j.neuroimage.2015.11.015 26569570 PMC4767224

[bib16] Friston K. J., Stephan K., Li B. J., & Daunizeau J. (2010). Generalised filtering. Mathematical Problems in Engineering, Article ID 621670 10.1155/2010/621670

[bib17] Kahan J., Urner M., Moran R., Flandin G., Marreiros A., Mancini L., … Foltynie T. (2014). Resting state functional MRI in Parkinson’s disease: The impact of deep brain stimulation on “effective” connectivity. Brain, 137(4), 1130–1144. 10.1093/brain/awu027 24566670 PMC3959559

[bib18] Li B., Daunizeau J., Stephan K. E., Penny W., Hu D., & Friston K. (2011). Generalised filtering and stochastic DCM for fMRI. NeuroImage, 58(2), 442–457. 10.1016/j.neuroimage.2011.01.085 21310247

[bib19] Margulies D. S., Ghosh S. S., Goulas A., Falkiewicz M., Huntenburg J. M., Langs G., … Smallwood J. (2016). Situating the default-mode network along a principal gradient of macro scale cortical organization. Proceedings of the National Acad emy of Sciences. 10.1073/pnas.1608282113 PMC5098630 27791099

[bib20] Nakagawa T. T., Jirsa V. K., Spiegler A., McIntosh A. R., & Deco G. (2013). Bottom up modeling of the connectome: Linking structure and function in the resting brain and their changes in aging. NeuroImage, 80, 318–329. 10.1016/j.neuroimage.2013.04.055 23629050

[bib33] Penny W. D., Friston K. J., Ashburner J. T., Kiebel S. J., & Nichols T. E. (2011). Statistical parametric mapping: The analysis of functional brain images. London: Academic Press.

[bib21] Qi S. L., Meesters S., Nicolay K., Romeny B. M. T., & Ossenblok P. (2015). The influence of construction methodology on structural brain network measures: A review. Journal of Neuroscience Methods, 253, 170–182. 10.1016/j.jneumeth.2015.06.016 26129743

[bib22] Raichle M. E. (2011). The restless brain. Brain Connect, 1(1), 3–12. 10.1089/brain.2011.0019 22432951 PMC3621343

[bib23] Raj A., Kuceyeski A., & Weiner M. (2012). A network diffusion model of disease progression in dementia. Neuron, 73(6), 1204–1215. 10.1016/j.neuron.2011.12.040 22445347 PMC3623298

[bib24] Razi A., & Friston K. J. (2016). The connected brain causality, models, and intrinsic dynamics. IEEE Signal Processing Magazine, 33(3), 14–35. 10.1109/Msp.2015.2482121

[bib25] Razi A., Kahan J., Rees G., & Friston K. J. (2015). Construct validation of a DCM for resting state fMRI. NeuroImage, 106, 1–14. 10.1016/j.neuroimage.2014.11.027 25463471 PMC4295921

[bib26] Rubinov M., & Sporns O. (2010). Complex network measures of brain connectivity: Uses and interpretations. NeuroImage, 52(3), 1059–1069. 10.1016/j.neuroimage.2009.10.003 19819337

[bib27] Rubinov M., & Sporns O. (2011). Weight-conserving characterization of complex functional brain networks. NeuroImage, (56), 4 2068–2079. 10.1016/j.neuroimage.2011.03.069 21459148

[bib34] Seghier M. L., & Friston K. J. (2013). Network discovery with large DCMs. NeuroImage, 68, 181–191. 10.1016/j.neuroimage.2011.03.069 23246991 PMC3566585

[bib28] Simpson S. L., Bowman F. D., & Laurienti P. J. (2013). Analyzing complex functional brain networks: Fusing statistics and network science to understand the brain. Statistics Surveys, 7, 1–36. 10.1214/13-SS103 25309643 PMC4189131

[bib29] Smith S. M., Vidaurre D., Beckmann C. F., Glasser M. F., Jenkinson M., Miller K. L., … Van Essen D. C. (2013). Functional connectomics from resting-state fMRI. Trends Cognitive Sciences, 17(12), 666–682. 10.1016/j.tics.2013.09.016 24238796 PMC4004765

[bib30] Sporns O. (2014). Contributions and challenges for network models in cognitive neuroscience. Nature Neuroscience, 17(5), 652–6660. 10.1038/nn.3690 24686784

[bib31] Sporns O., & Betzel R. F. (2016). Modular brain networks. Annual Review of Psychology, 67, 613–640. 10.1146/annurev-psych-122414-033634 PMC4782188 26393868

